# Protocol for a randomized controlled trial: efficacy and mechanisms of dual-target anodal tDCS in post-stroke cognitive impairment

**DOI:** 10.3389/fnhum.2025.1603797

**Published:** 2025-08-13

**Authors:** Xiayan Xue, Sicong Zhang, Qingjuan Guo, Jiali Wu, Jingjing Zhang, Cong Wang, Chunlei Shan

**Affiliations:** ^1^Department of Rehabilitation Medicine, Yueyang Hospital of Integrated Traditional Chinese and Western Medicine, Shanghai University of Traditional Chinese Medicine, Shanghai, China; ^2^School of Rehabilitation Science, Shanghai University of Traditional Chinese Medicine, Shanghai, China; ^3^Rehabilitation Center, Tongren Hospital, Shanghai Jiao Tong University School of Medicine, Shanghai, China; ^4^Shanghai Key Laboratory of Flexible Medical Robotics, Tongren Hospital, Institute of Medical Robotics, Shanghai Jiao Tong University School of Medicine, Shanghai, China; ^5^Yuanshen Rehabilitation Institute, Shanghai Jiao Tong University School of Medicine, Shanghai, China

**Keywords:** post-stroke cognitive impairment, transcranial direct current stimulation, dual-target stimulation, dorsolateral prefrontal cortex, angular gyrus, functional near-infrared spectroscopy

## Abstract

**Background:**

Post-stroke cognitive impairment (PSCI) significantly hinders functional recovery and quality of life in stroke survivors. Although transcranial direct current stimulation (tDCS) has emerged as a promising non-invasive neuromodulation technique to improve cognitive function, conventional single-target tDCS approaches often yield inconsistent outcomes across cognitive domains.

**Methods:**

This randomized controlled trial aims to investigate the efficacy and underlying neural mechanisms of dual-target anodal tDCS (a-tDCS) in individuals with PSCI. Sixty participants will be randomly assigned to either a dual-target stimulation group or a single-target stimulation group. Each group will receive 10 sessions of tDCS over two weeks. Cognitive performance will be assessed using the Montreal Cognitive Assessment (MoCA) as the primary outcome, while secondary outcomes include the digit span test, Trail Making Test, and modified Barthel Index. Functional near-infrared spectroscopy (fNIRS) will be used to assess cortical activation and functional connectivity before and after the intervention.

**Discussion:**

The findings are expected to provide evidence on the efficacy and underlying mechanisms of dual-target tDCS in PSCI rehabilitation, potentially offering a more effective neuromodulatory intervention strategy for cognitive recovery in stroke survivors.

**Clinical trial registration:**

http://www.chictr.org.cn, identifier ChiCTR2500096896.

## Introduction

### Background and rationale

Stroke is a neurological disorder caused by disruptions in cerebral blood flow, and it remains the second leading cause of death worldwide, with more than 13 million new cases reported annually (Feigin et al., 2022). According to the Global Burden of Disease Study 2019, China recorded 3.94 million new stroke cases, 28.76 million prevalent cases, and 2.19 million stroke-related deaths that year. Stroke also accounted for the highest number of disability-adjusted life years in the country, totaling 45.9 million in 2019 (Wang et al., 2022). This has led to China having the highest lifetime risk and the heaviest disease burden of stroke globally (GBD 2016 Causes of Death Collaborators, 2017).

Post-stroke cognitive impairment (PSCI) affects up to 1/3 of stroke survivors and significantly impairs their ability to engage in daily activities and social participation (Wang et al., 2021). Despite its high prevalence, awareness of PSCI remains low. One study in China found that only 39.1% of patients knew about the risk of post-stroke dementia, and only 8.8% sought medical help for cognitive decline (Zhou et al., 2021). PSCI is associated with worse recovery outcomes (Sloane and Hamilton, 2024). Cognitive processes play a crucial role in post-stroke learning across modalities, and motor performance is particularly influenced by cognitive function, especially as task complexity increases (Chen et al., 2013; Mullick et al., 2015). Studies show that impairments in memory and executive function are linked to motor deficits, and worse cognitive status leads to poorer responsiveness to rehabilitation interventions (Einstad et al., 2021). PSCI presents a major burden for both individuals and healthcare systems, and improving cognitive function in stroke survivors is a critical goal for rehabilitation.

Current treatments for PSCI, including pharmacological interventions and cognitive rehabilitation, have limitations. Medications such as acetylcholinesterase inhibitors, memantine, and sodium oligomannate (Huang et al., 2022) may provide short-term benefits, but there is little evidence to suggest they can prevent further cognitive decline or restore function in stroke survivors (Liu et al., 2024). Long-term medication use is often associated with side effects (Wang et al., 2021) which limits their feasibility as a long-term solution. Rehabilitation programs, on the other hand, are time-intensive and yield variable outcomes (Zhou et al., 2024). Therefore, there is a pressing need for more effective, non-invasive interventions to support cognitive recovery.

Non-drug treatments, particularly non-invasive brain stimulation, are gaining attention as promising alternatives. Transcranial direct current stimulation (tDCS) is a non-invasive neuromodulation technique that modulates neuronal excitability, with anodal stimulation increasing cortical excitability and cathodal stimulation reducing it (Nitsche and Paulus, 2000). It has been shown that tDCS is effective in promoting neuroplasticity and enhancing cognitive function. Studies have demonstrated its potential in PSCI, showing improvements in various cognitive domains, including global cognition (Cappon et al., 2016), memory (Jm et al., 2009; Yun et al., 2015; Kazuta et al., 2017), and executive function (Hosseinzadeh et al., 2018).

Despite its promise, traditional tDCS still has limitations in treating PSCI. The therapeutic effects show significant heterogeneity, particularly in domains like attention and memory, where improvements are often inconsistent. While tDCS demonstrates efficacy in enhancing executive and visuospatial functions, its impact on memory and attention remains limited (Zhao et al., 2024). To address this gap, further research is needed to optimize traditional tDCS and explore advanced strategies. Dual-target tDCS may be one of the potential approaches. Dual-target tDCS offers a potential solution by simultaneously modulating excitatory and inhibitory activity in both hemispheres. A study (Li et al., 2023) places the anodal electrode over the ipsilesional primary somatosensory cortex (PSC) to enhance excitability and the cathodal electrode over the contralesional PSC to suppress overactivation. In subacute stroke patients, this approach improved motor and sensory functions. Functional near-infrared spectroscopy (fNIRS) showed improved connectivity between the ipsilesional supplementary motor cortex and contralesional sensory association cortex, suggesting bilateral neural network remodeling.

While dual-target tDCS with opposite polarities has shown efficacy in motor recovery (Li et al., 2023), its potential for cognitive rehabilitation remains underexplored. One study reported dual-target tDCS effectively improved specific cognitive domains, including attention and concentration, figural memory, logical reasoning, and reaction behavior (Shaker et al., 2018). However, another study focusing on stop-signal tasks found no consistent improvements in performance using dual-target tDCS with opposite polarities (Friehs et al., 2021). Due to the reduction of corticospinal excitability under the cathode, some researchers remain doubtful about the applicability of this approach for cortical or behavioral modifications (Vaseghi et al., 2015).

Therefore, a new approach, dual-target anodal tDCS (a-tDCS) has been proposed, demonstrating significant effects in motor performance enhancement. In healthy adults, it significantly enhances corticospinal excitability in the motor cortex, with effects approximately twice as strong as conventional methods and lasting for at least 24 h. This technique may promote long-term brain plasticity by enhancing glutamatergic and NMDA receptor-dependent mechanisms, offering promising applications in motor recovery and neurorehabilitation (Vaseghi et al., 2015). In subacute stroke patients, dual-target a-tDCS improves motor function by reducing reaction time, enhancing hand dexterity, and increasing corticospinal excitability while strengthening connectivity between the DLPFC and primary motor cortex (Achacheluee et al., 2018). In the cognitive domain, dual-target a-tDCS remains underexplored. However, previous studies on dual-target high-definition tDCS (HD-tDCS) provide valuable insights. A recent study showed that dual-target anodal HD-tDCS significantly improved response inhibition in healthy participants (Guo et al., 2022).

Despite its potential, HD-tDCS is complex and costly, posing challenges for widespread application. If a simpler, more affordable method like dual-target a-tDCS can demonstrate similar effectiveness, it could enable broader access to neuromodulation interventions, allowing more patients to benefit from it. This highlights the importance of further investigation into dual-target a-tDCS as a potential alternative in PSCI.

The dorsolateral prefrontal cortex (DLPFC) and angular gyrus (AG) are two critical brain regions implicated in cognitive processes. DLPFC is an important target for brain stimulation because it plays a key role in cognitive functions like memory, attention, and decision-making. It is part of the central executive network, which helps manage and coordinate complex mental tasks. Stimulating the DLPFC can increase brain activity, improve connections with other brain areas like the caudate nucleus, and support recovery of thinking abilities, making it a valuable focus for treating cognitive problems after a stroke (Han et al., 2023). The AG located in the posterior part of the inferior parietal lobule at the junction of the parietal, temporal, and occipital lobes, acts as a crucial convergence zone for multisensory and conceptual integration. It is an important hub in several neural networks, including the Default Mode Network for memory retrieval and self-referential processing, the Central Executive Network for working memory and attention control, and the Cross-Modal Integration Network for sensory-conceptual integration (Seghier, 2013). Functionally, the AG supports higher-order cognitive tasks including semantic processing, episodic memory retrieval, attentional regulation, numerical reasoning, spatial cognition, and social understanding, bridging perception with advanced cognitive processes (Seghier, 2023). In Alzheimer’s disease and mild cognitive impairment (Yang et al., 2022), stimulation of the AG has been shown to improve cognitive functions.

This trial aims to evaluate the clinical efficacy of dual-target a-tDCS targeting the DLPFC and AG in patients with PSCI and to investigate the underlying neural mechanisms using fNIRS. We hypothesize that dual-target a-tDCS will outperform single-target a-tDCS in improving cognitive function and enhancing brain connectivity.

### Objectives

#### Primary objective

The primary objective of this randomized controlled trial is to evaluate the clinical efficacy of dual-target a-tDCS in improving cognitive function in patients with PSCI. This will be assessed by comparing cognitive improvements between two tDCS interventions: (1) a-tDCS stimulation of the DLPFC alone, and (2) dual-target a-tDCS stimulation of the DLPFC and the AG. We hypothesize that dual-target stimulation will lead to greater improvements in overall cognitive function, including memory and executive function, compared to single-target stimulation.

#### Secondary objectives

The secondary objectives are to investigate the neural mechanisms underlying the effects of these tDCS interventions using fNIRS. Specifically, we aim to:

(1)  Assess changes in brain activation in the DLPFC and AG regions.
(2)  Evaluate the impact of dual-target a-tDCS on functional connectivity between cognitive networks associated with PSCI.
(3)  Explore the correlation between changes in brain activity and cognitive improvements observed in PSCI patients.

### Trial design

**FIGURE 1:**
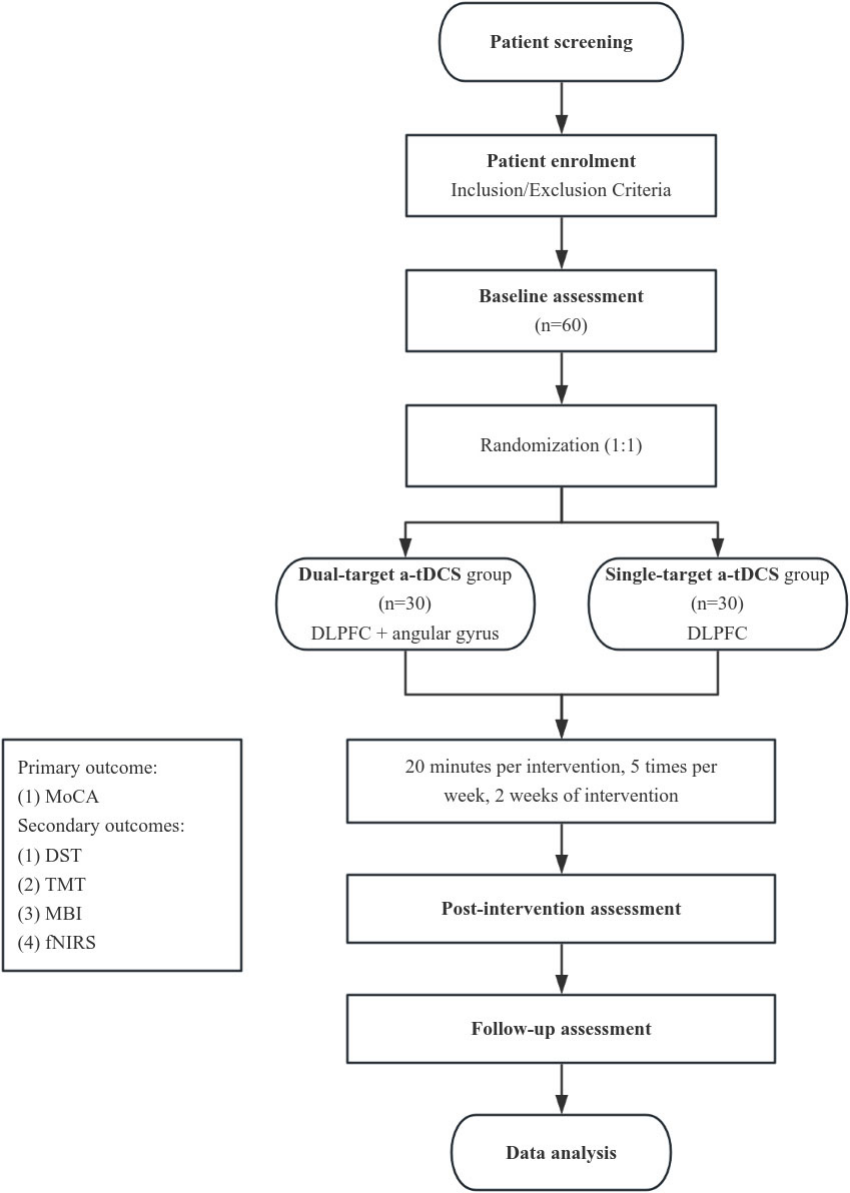
Study flowchart. Overview of the randomized controlled trial design, including participant screening, randomization, intervention sessions (dual-target vs. single-target a-tDCS), and outcome assessments at pre-intervention, post-intervention, and follow-up stages.

This study is a single-center, participant-blinded, parallel-group, randomized controlled trial with a 1:1 allocation ratio. The trial is designed as an exploratory study to investigate the effects of dual-target a-tDCS compared to single-target a-tDCS on cognitive functions of patients with PSCI. Participants will be blinded to the intervention they receive, while the operators administering the tDCS will not be blinded due to the nature of the intervention. Outcome assessors and data analysts will remain blinded to group allocation to reduce bias during the evaluation and analysis phases. A detailed overview of the study design and participant flow is illustrated in Figure 1. This study is designed as a comparative trial between two active tDCS conditions (dual-target and single-target). A sham control group is not included due to ethical considerations and the aim to compare different active stimulation protocols rather than to test tDCS efficacy against placebo.

## Methods: participants, interventions, and outcomes

### Study setting

This study will be conducted at a single site, Yueyang Hospital of Integrated Traditional Chinese and Western Medicine, Shanghai University of Traditional Chinese Medicine. Yueyang Hospital is an academic tertiary care hospital specializing in stroke rehabilitation and cognitive impairment research.

Data collection will occur exclusively at this site in Shanghai, China. For further information on the study site, or to obtain a list of collaborating institutions if applicable, please refer to the corresponding section on the Chinese Clinical Trial Registry or contact the principal investigator.

### Eligibility criteria

#### Inclusion criteria

(1)  Confirmed Diagnosis of Stroke: Patients aged 18–80 years with a confirmed diagnosis of ischemic or hemorrhagic stroke, as evidenced by clinical and imaging findings.
(2)  PSCI: Participants must have a Montreal Cognitive Assessment (MoCA) score less than 26, indicating cognitive impairment following stroke.
(3)  Time Since Stroke: The stroke must have occurred at least 1 month prior to enrollment, and the patient’s condition should be stable.
(4)  Language Proficiency: Participants must be native speakers of Chinese with no significant aphasia, enabling them to complete cognitive assessments.
(5)  Informed Consent: Participants must be willing and able to provide written informed consent to participate in the study.

#### Exclusion criteria

(1)  Progressive Cerebrovascular Disease: Patients with evidence of progressive stroke or worsening cerebrovascular disease.
(2)  Severe Medical Conditions: Presence of severe cardiac, hepatic, or renal diseases that could interfere with participation or endanger the participant’s health.
(3)  History of Epilepsy: Patients with a history of epilepsy or a family history of epilepsy, or those who have recently used epileptogenic medications.
(4)  Previous Neurological Surgery or Severe Head Injury: History of skull fractures, severe head trauma, or previous brain surgery.
(5)  Substance Abuse and Pregnancy: Patients with a history of substance abuse (e.g., smoking, alcoholism) or who are currently pregnant.

### Eligibility criteria for personnel performing interventions

Interventions will be performed by licensed neurologists and trained therapists with experience in administering tDCS. All personnel must have undergone specific training in the study protocol and cognitive assessments used in the trial.

#### Informed consent

This trial aims to evaluating the clinical efficacy of dual-target a-tDCS in patients with PSCI, and to investigate the underlying neural mechanisms using fNIRS. We hypothesize that dual-target a-tDCS will be more effective than single-target stimulation in improving cognitive function and enhancing brain connectivity.

Informed consent will be distributed by the study’s principal investigator (PI) or a designated member of the research team, who has been thoroughly trained in the ethical principles and procedures of obtaining consent. The individuals responsible for obtaining consent will be experienced in interacting with stroke patients and their families, ensuring they can effectively communicate the study’s details.

### Additional consent provisions for collection and use of participant data and biological specimens

This trial does not involve collecting biological specimens.

### Interventions

#### Explanation for the choice of comparators

For this study, single-target a-tDCS will be used as the comparator which has been widely studied and shown to be effective in modulating neuronal activity and improving cognitive function in various neurorehabilitation settings. However, its effects on PSCI are limited. The rationale for selecting single-target a-tDCS as the comparator lies in its established role in promoting cortical excitability and neuroplasticity, serving as a suitable standard intervention against which the potential added benefits of dual-target a-tDCS can be evaluated. The use of single-target a-tDCS allows us to directly compare whether stimulating two brain regions simultaneously results in superior cognitive outcomes and brain connectivity improvements compared to traditional single-site stimulation. To minimize potential confounding, no additional formal cognitive training is included during the 2-week tDCS treatment period. Usual care (e.g., physical or occupational therapy) is allowed if not cognitively targeted. The tDCS sessions are not combined with concurrent cognitive tasks. The stimulation is administered at rest in order to examine the independent effects of tDCS on cognitive function.

#### Intervention description

Participants in the intervention group will receive dual-target a-tDCS, targeting both the DLPFC and the AG. Each session will be administered once daily, 5 days per week, for a total of 10 sessions over 2 weeks. The stimulation schedule of 5 sessions per week for 2 weeks (10 sessions in total) is adopted with reference to established clinical practice and evidence-based recommendations. According to the international guideline on therapeutic tDCS use (Lefaucheur et al., 2017), most effective protocols involve repeated daily stimulation over 1–4 weeks, with 10–20 total sessions commonly used across a range of neurological and psychiatric conditions. In the context of stroke rehabilitation, previous studies have typically applied between 5 and 20 tDCS sessions depending on treatment goals and population characteristics. In addition, a recent component network meta-analysis (Chu et al., 2021) reported that tDCS protocols applied daily for 2–5 weeks were commonly used in patients with Alzheimer’s disease and mild cognitive impairment, supporting the acceptability and cognitive efficacy of repeated daily stimulation schedules.

For post-stroke subpopulations, 10-session regimens delivered as five sessions per week over 2 weeks are widely applied in aphasia studies (Biou et al., 2019), and similar protocols have been used for patients with PSCI. For example, this schedule was applied in PSCI patients and reported significant behavioral and neurophysiological improvements (Ai et al., 2024). These findings support the feasibility and effectiveness of our chosen stimulation schedule. The tDCS sessions will be delivered using a transcranial electrical stimulation (tES) device Soterix Medical 1X1 Transcranial Electrical Low-Intensity Stimulator, Model 2001 with a current intensity of 1.5 mA. Anodal electrodes will be placed over the DLPFC (F3/F4) and AG (P3/P4), while cathodal electrodes will be placed on the contralateral shoulder. Each session will last 20 min, during which participants will be seated comfortably, and skin preparation will include cleaning the electrode sites to ensure good contact.

In the control group, participants will receive single-target tDCS, targeting only the DLPFC. The parameters for this group will be identical to those for the intervention group, except that the AG will not be stimulated. Instead, sham stimulation will be applied to the AG, simulating the sensation of tDCS without delivering an active current. Set the SHAM switch to ON, enabling the sham mode. During this mode, the current ramps up to the set intensity over a period of 30 s at the beginning of the session and ramps down in 30 s. No stimulation is delivered during the interim period. This ramp-up and ramp-down process is repeated at the end of the session to mimic the sensory perception of active stimulation without inducing neuromodulatory effects. This approach will help maintain blinding between the groups.

#### Criteria for discontinuing or modifying allocated interventions

Interventions will be discontinued or modified if participants experience significant adverse reactions, such as severe headaches or skin irritation. In such cases, the session will be paused, and the participant will be closely monitored. If the symptoms persist, the intervention may be discontinued, or the current intensity may be reduced. Participants who wish to withdraw from the study or request modifications to the intervention due to discomfort or personal reasons will be allowed to do so without penalty.

If a participant’s condition worsens during the trial, such as experiencing a new stroke event or a decline in cognitive function, the intervention will be reviewed and may be modified or discontinued based on the situation.

#### Strategies to improve adherence to interventions

To improve adherence, participants will be regularly monitored by study physicians, who will stay in contact to encourage their progress. Automated reminders will be sent via phone or text before each session to ensure timely attendance. Compliance will be tracked by recording attendance, and any missed session will be followed up promptly to reschedule.

#### Relevant concomitant care permitted or prohibited during the trial

Participants are allowed to continue their standard care during the trial, including prescribed medications and routine physical therapy. However, any additional neuromodulation interventions (e.g., other forms of brain stimulation) are prohibited to avoid confounding effects. Participants will be instructed to avoid any new cognitive rehabilitation programs outside the study during the trial period to ensure the study’s integrity.

#### Provisions for post-trial care

If any adverse events occur during the clinical trial, a medical expert committee will assess the relationship between the event and the trial protocol. If it is determined that the adverse event is related to the trial, the research team will cover the cost of treatment for any harm caused by the trial and provide appropriate financial compensation.

### Outcomes

#### Primary outcomes

The MoCA is a cognitive screening tool that evaluates multiple domains, including memory, attention, language, and executive function. It has proven to be highly reliable and valid in stroke patients, with a test-retest reliability of 0.96 and inter-rater reliability of 0.87–0.95 in Chinese versions. The MoCA is more sensitive than the mini-mental state examination (MMSE) for detecting cognitive impairment in stroke patients (Zuo et al., 2024), making it an ideal choice for assessing post-stroke cognitive deficits.

#### Secondary outcomes

We will use digit span test (DST) to evaluate the ability of memory, trail making test (TMT) to evaluate the ability of attention (Nishimura et al., 2022), memory (Slavin et al., 2022), and executive function. The modified Barthel index (MBI) will be used to assess activities of daily living (Wang et al., 2023).

**FIGURE 2:**
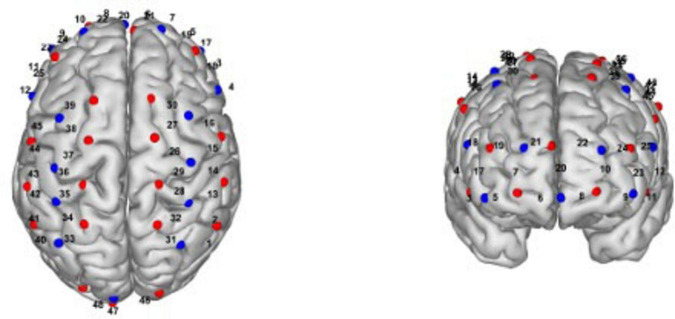
Spatial layout of fNIRS channels. The figure illustrates the spatial configuration of the fNIRS channels projected onto a standard head model.

Additionally, we will collect fNIRS data using a multichannel fNIRS system (NirSmart, Danyang Huichuang Medical Equipment, China), which is a non-invasive optical neuroimaging technique that enables real-time monitoring of changes in oxygenated hemoglobin (HbO2), deoxygenated hemoglobin (HbR) (Ning et al., 2024), and cortical hemodynamics in the cerebral cortex. fNIRS is selected as the neuroimaging method in this study due to its practical and methodological advantages over other commonly used techniques such as functional magnetic resonance imaging (fMRI) and electroencephalography (EEG). Although fMRI is regarded as the gold standard for assessing brain activity, previous studies have demonstrated a strong correlation between fMRI BOLD signals and the hemodynamic responses captured by fNIRS, indicating comparable capacity in detecting cortical blood oxygen changes (Cui et al., 2011; Sato et al., 2013). However, fMRI requires participants to remain immobilized in a confined and noisy environment, which may not be feasible for stroke patients, especially those with PSCI, who may have difficulty maintaining stillness or lying supine for extended periods. In contrast, fNIRS offers higher tolerance to motion artifacts, greater portability, lower cost, and more flexible experimental conditions, making it well suited for repeated assessments in naturalistic and rehabilitation settings (Sui et al., 2022; Fu et al., 2023; Zou et al., 2023). Besides, fNIRS has higher temporal resolution than fMRI (Zou et al., 2023). Compared to EEG, fNIRS is less affected by electrical noise and muscle artifacts (Fu et al., 2023). Importantly, fNIRS can be used during ecologically valid cognitive tasks performed in a seated position. In our study, participants are required to complete the TMT, which requires sitting in front of a table and drawing lines by hand. fNIRS enables real-time monitoring of cortical hemodynamics during tasks without interfering with performance. These features make fNIRS an ideal tool for assessing the neurophysiological effects of tDCS in PSCI rehabilitation. The system uses dual wavelengths (730 nm and 850 nm) and consists of 16 detectors and 24 LED emitters, resulting in 48 channels with an average source-detector distance of 30 mm, and a sampling frequency of 11 Hz. To assess functional connectivity (FC), a 10-min resting-state fNIRS measurement will be performed to examine pre- and post-intervention changes. For task-state fNIRS, participants will wear the fNIRS headcaps while completing the TMT, allowing us to simultaneously capture cerebral hemodynamic changes during the task. A spatial layout of the fNIRS channels is presented in Figure 2.

#### Participant timeline

Participants will follow a structured timeline throughout the study, consisting of enrollment, intervention sessions, assessments, and follow-up visits.

At the enrollment visit (Week 0), participants will undergo initial screening to confirm eligibility. Baseline assessments will include the MoCA, DST, TMT, MBI, and fNIRS parameters.

During the intervention period (Weeks 1–2), participants will receive 10 sessions of tDCS over 2 weeks, with 5 sessions per week. At the end of Week 2, immediately after the final tDCS session, participants will undergo post-intervention assessments using the same measures as at baseline, including MoCA, DST, TMT, MBI, and fNIRS. For follow-up (Week 14), participants will return 3 months after the last tDCS session for follow-up assessments using the same battery of measures to monitor long-term effects on cognitive function and brain activity. The SPIRIT plan is shown in Table 1.

**TABLE 1:** SPIRIT plan.

	Study period				
Timepoint	Enrolment	Allocation	Post-allocation		Follow-up
		t0	t1	t2	t3
**Enrolment**					
Eligibility screen	√				
Informed consent	√				
Demographic information	√				
Allocation	√	√			
**Interventions**					
Dual-target group			√	√	
Single-target group			√	√	
**Assessments**					
MoCA		√		√	√
Digit span		√		√	√
TMT		√		√	√
MBI		√		√	√
fNIRS		√		√	√

### Sample size

The primary outcome measure for this trial is the MoCA score after 10 treatment sessions. The sample size estimation was conducted using a two-sample means comparison. Based on similar published studies (Gonzalez et al., 2021), it is known that the mean MoCA scores after treatment in the experimental group and the control group are assumed to be 25.7 and 23.7, with standard deviations of 2.4 and 1.7, respectively. With a significance level of α = 0.05 (two-sided test) and a power of 1-β = 0.9, assuming equal sample sizes for the experimental and control groups, the following formula was used:


n=(Z1-α/2+Z1-β)2×(σ12+σ22)δ2

In the formula: Z_1−α/2_ = 1.96, Z_1−β_ = 1.28, σ_1_ and σ_2_ are the pooled standard deviation of the two groups, and δ is the difference between the two group means.

Using the “Two-Sample *T*-Tests Allowing Unequal Variance” option in PASS 2021 software, the calculation determined that each group requires 24 subjects. Considering a dropout rate of 20%, 30 subjects will be enrolled in both the experimental and control groups, totaling 60 subjects, to ensure the scientific rigor of the study design.

### Recruitment

The target population for this study consists of patients with PSCI who meet the eligibility criteria and are receiving treatment at Yueyang Hospital of Integrated Traditional Chinese and Western Medicine, Shanghai University of TCM. Recruitment will be carried out through collaboration with stroke rehabilitation units within the hospital, where neurologists and rehabilitation specialists will identify potential participants during routine clinical visits. Patients who meet the inclusion criteria will be approached by the study team and provided with detailed information about the trial. The hospital’s electronic medical records system will be used to identify stroke patients, and existing stroke patient registries will also be utilized. Recruitment posters at the clinic and website recruitment advertisements will be used.

### Assignment of interventions: allocation

#### Sequence generation

After the baseline assessment, participants will be randomly assigned to one of two groups in a 1:1 ratio using computer-generated random numbers.

#### Concealment mechanism

The allocation sequence will be concealed using sequentially numbered, opaque, sealed envelopes to ensure that the allocation remains hidden until interventions are assigned. Each envelope will contain a card indicating the group assignment (intervention or control) and will be opened sequentially only after a participant has been enrolled and baseline assessments have been completed.

The envelopes will be prepared in advance by a research assistant who is not involved in the enrollment or intervention process. The envelopes will be stored in a secure location with restricted access to prevent any potential tampering or accidental disclosure of the allocation sequence.

To further enhance the concealment, the envelopes will be identical in appearance, and the sequence number will be printed on the outside of the envelope without any indication of the group assignment inside.

#### Implementation

The allocation sequence will be generated by an independent biostatistician who is not involved in participant enrollment, intervention delivery, or outcome assessment. A computer-generated randomization program will be used, stratifying participants by age and baseline cognitive function to ensure balance between groups.

Participants will be enrolled by study clinicians who are responsible for screening and performing baseline assessments. The clinicians involved in enrollment will not have access to the allocation sequence or any information about group assignments.

After a participant has been enrolled and completed their baseline assessments, the study clinician will contact a research assistant to retrieve the next sequentially numbered, opaque, sealed envelope. The envelope will be opened in the participant’s presence, and the group assignment will be revealed. The research assistant, blinded to the allocation sequence, will assign the participant to the corresponding intervention group.

### Assignment of interventions: blinding

#### Who will be blinded

Participants will be blinded to their group assignment (intervention or control). They will not know whether they are receiving active dual-target a-tDCS or sham stimulation, ensuring that their expectations do not influence the outcomes.

The therapists administering the tDCS will not be blinded to the group assignments. They will know whether they are providing active or sham stimulation due to the nature of the intervention.

Outcome assessments will be conducted by clinicians who are blinded to the participants’ group assignments. Each participant will be identified by a unique study number, and the outcome data will be recorded without revealing the group allocation.

Data analysis will be performed by an independent data analyst who will be blinded to the group assignments. The analyst will work with de-identified data, ensuring that the analysis remains unbiased.

#### Procedure for unblinding if needed

Unblinding will only be considered in specific circumstances where it is deemed essential for the participant’s safety, such as in the case of a serious adverse event that requires knowledge of the treatment received for appropriate medical intervention.

### Data collection and management

#### Plans for assessment and collection of outcomes

All assessors will undergo rigorous training in the administration of cognitive assessments and fNIRS measurements to ensure consistency and accuracy. Standardized operating procedures will be developed and followed for all data collection processes, including detailed instructions on administering each assessment tool. All data collection forms will be available upon request and will be stored securely within the study’s data management system.

#### Plans to promote participant retention and complete follow-up

To enhance participant adherence, a research assistant will send reminders via phone calls and text messages to ensure they attend all scheduled assessments and intervention. If participants discontinue or deviate from intervention protocols, the study team will initiate contact and prioritize addressing any concerns that may be impacting their adherence to the intervention protocols. Transportation reimbursements will be provided to encourage participants to complete the study.

#### Data management

All data collected during the trial, including baseline, intervention, and follow-up assessments, will be entered into a secure electronic database using the China Clinical Trial Registration Center’s electronic data management platform (ResMan Research Manager). Data entry will be performed by trained personnel following standardized protocols to ensure accuracy and consistency. To minimize data entry errors, a double data entry process will be employed, where two independent data entry personnel will input the data separately. Any discrepancies between the two entries will be resolved through a verification process by a third party. All participant data will be anonymized using unique participant identification codes, replacing personal identifiers to maintain confidentiality. Hard copies of any paper-based data collection forms will be stored in locked filing cabinets within a restricted access area at the study site to ensure data security.

#### Confidentiality

This trial will follow the Personal Information Protection Law of China. The database will be kept confidential and anonymous. Personal information about potential and enrolled participants will be collected only by authorized members of the research team. Only the minimum necessary information will be collected to achieve the trial’s objectives, which includes demographic data, medical history, contact details, and any other relevant information necessary for the conduct of the trial. All personal information will be stored securely. Personal identifiers, such as names and contact information, will be removed or replaced with unique participant codes in the main study database. No study details can be disclosed to unauthorized third par-ties without prior approval.

#### Plans for collection, laboratory evaluation and storage of biological specimens for genetic or molecular analysis in this trial/future use

Not applicable. No specimens will be collected.

### Statistical methods

#### Statistical methods for primary and secondary outcomes

The primary outcome of this study is the change in MoCA scores from baseline to post-intervention and follow-up. The changes in MoCA scores from baseline to post-tDCS intervention, and during the follow-up assessment, will be compared between the experimental and control groups. Homogeneity of variance will be assessed by Levene’s test. For normally distributed continuous data, ANOVA will be used to compare differences between the two groups. If ANOVA indicates significant differences, further post-hoc analysis will be performed using Bonferroni correction. For non-normally distributed continuous data, the Wilcoxon test will be used. The significance level (α) for statistical hypothesis testing will be set at *P* < 0.05. Differences with *P* < 0.05 will be considered statistically significant, while those with *P* > 0.05 will be considered not statistically significant.

Secondary outcomes, including changes in DST, TMT-A and TMT-B, and MBI scores, will be analyzed using similar repeated measures ANOVA methods. For non-normally distributed continuous data, the Wilcoxon signed-rank test will be used for within-group comparisons, and the Mann-Whitney U test will be used for between-group comparisons. fNIRS data will be analyzed using mixed-effects models to assess changes in cortical activation and connectivity, accounting for random effects of participants and fixed effects of group and time. Correlation analysis, such as Pearson correlation coefficient or Spearman correlation coefficient will be conducted to explore the relationships between primary and secondary outcome measures. Lesion location (cortical/subcortical), stroke subtype (ischemic/hemorrhagic), hemispheric lateralization (left/right) and stroke phase (subacute/chronic) will be recorded based on imaging reports. Exploratory analyses may include lesion location and laterality as covariates if appropriate, to explore their influence on intervention outcomes.

#### Interim analyses

Data will be performed at any interim analysis only by blinded data analysts, and the trial steering committee will have access to these interim results and make the final decision to terminate the trial.

#### Methods for additional analyses (e.g., subgroup analyses)

Subgroup analyses are not planned for this study.

#### Methods in analysis to handle protocol non-adherence and any statistical methods to handle missing data

The primary analysis will follow an intention-to-treat (ITT) approach, including all participants as randomized, regardless of protocol adherence.

Missing data will be addressed using multiple imputation methods, assuming data are missing at random. Imputation models will include baseline characteristics, group assignment, and observed outcome values.

Sensitivity analyses will be conducted to assess the robustness of the results under different missing data assumptions, including complete case analysis and worst-case scenario imputation.

#### Plans to give access to the full protocol, participant level-data and statistical code

The datasets of this study are available on the clinical trial registration website.

### Oversight and monitoring

#### Composition of the coordinating center and trial steering committee

A Trial Steering Committee (TSC) will be established to ensure quality assurance, oversee protocol amendments, monitor the conduct of the study, and assess outcomes. The TSC will meet regularly to closely supervise trial progress. It will have unblinded access to study data and will determine whether to continue the trial based on assessments of safety, efficacy, and compliance.

#### Composition of the data monitoring committee, its role and reporting structure

Neurologists, methodologists, and statisticians will supplement the data monitoring committee (DMC). The DMC will be responsible for overseeing data quality management and safety monitoring. DMC meetings will be held regularly. Clinical Trial Data Management issued by the Chinese National Medical Products Administration.

#### Adverse event reporting and harms

Participants experiencing adverse events (AEs) will be provided with appropriate medical care. If necessary, the intervention will be paused or discontinued based on the clinical judgment of the research team and in consultation with the DMC. A specific protocol will be in place for managing skin irritation or discomfort associated with the tDCS, including immediate cessation of stimulation and application of soothing treatments.

All AEs will be documented in the participant’s case report form (CRF) and followed until resolution or stabilization. Follow-up assessments will be conducted to monitor the participant’s recovery or the persistence of symptoms.

#### Frequency and plans for auditing trial conduct

A dedicated, qualified individual will conduct and monitor this trial. The steering committee will meet regularly to review and evaluate updates.

#### Plans for communicating important protocol amendments to relevant parties (e.g., trial participants, ethical committees)

If the protocol changes during the implementation of the study, a revised protocol will be submitted for approval to the IRB of Yueyang Hospital of Integrated Traditional Chinese and Western Medicine, Shanghai University of TCM.

#### Dissemination plans

We will disseminate the study’s results through conference presentations and publications.

## Discussion

The DLPFC is pivotal in multiple cognitive processes, such as working memory, planning, inhibition, and abstract reasoning. Previous studies indicate that stimulation over the DLPFC can effectively improve cognitive deficits in stroke patients (Shaker et al., 2018; Cha et al., 2022). The AG also plays a vital role in various cognitive functions, including attention, spatial cognition, mental arithmetic, visuospatial processing, inhibitory control, theory of mind, and the processing of semantic information (Bonner et al., 2013; Grob et al., 2024; Park et al., 2024). Stimulation of the AG could enhance autobiographical memory integration (Bonner et al., 2013) and improve associative memory performance (Wang et al., 2014).

Building on these findings, this study hypothesizes that dual-target a-tDCS, focusing on both the DLPFC and AG, will be more effective in improving cognitive function in patients with PSCI compared to single-target a-tDCS on DLPFC. We anticipate significant enhancements in overall cognitive performance, specifically in domains like memory and executive function. Furthermore, we expect that fNIRS measurements will reveal increased activation in the DLPFC and AG and enhanced functional connectivity between cognitive brain networks.

If these hypotheses are confirmed, dual-target a-tDCS could become an effective intervention to improve cognitive outcomes in PSCI patients, potentially exceeding the benefits of conventional single-target interventions. This approach may be integrated into existing rehabilitation programs, thereby contributing to better recovery and an improved quality of life for stroke survivors.

However, the study has several limitations. The sample size is relatively small, and it is a single-center study, which may affect the generalizability of the results. The lack of long-term follow-up means that we could not assess the long-term effects of the intervention. Future research should aim to overcome these limitations by conducting multi-center trials with larger cohorts and longer follow-up periods.

Further exploration is necessary to assess its efficacy across different types and severities of PSCI.

The absence of a dual sham stimulation group may limit the interpretation of tDCS-specific effects versus non-specific intervention effects. Future studies may consider including a dual sham group to further strengthen causal inferences.

Inclusion of both subacute and chronic stroke patients may increase heterogeneity in treatment response. However, this reflects real-world practice, and the planned analyses will explore potential moderating effects of disease phase.

Although some studies suggest combine tDCS and cognitive training may enhance effects, we delivered tDCS alone to isolate its specific contribution and reduce procedural variability in a clinical rehabilitation context.

Additionally, further studies should employ more brain imaging techniques, including MRI and EEG, to comprehensively study the underlying neural mechanisms and better understand how dual-target a-tDCS affects brain connectivity and function.

This study introduces a novel dual-target a-tDCS protocol targeting both the DLPFC and AG to treat PSCI. It aims to fills a gap in the current literature and provides preliminary evidence for a potentially more effective intervention strategy for cognitive rehabilitation in stroke patients.

## Data Availability

Publicly available datasets were analyzed in this study. This data can be found here: not applicable.
